# PReS13-SPK-1586: Biological agents for the treatment of rheumatic diseases: present and future targets of biologic therapy

**DOI:** 10.1186/1546-0096-11-S2-I26

**Published:** 2013-12-05

**Authors:** I Mcinnes

**Affiliations:** 1 Institute of Infection, Immunity and Inflammation, University of Glasgow, Glasgow, UK

## 

The last decade has seen the advent of a variety of novel biologic agents, which are now used widely across a range of the inflammatory rheumatic diseases. Normally introduced after the trial and failure of conventional disease modifying anti-rheumatic drugs (DMARDs) the biologic class of medicines has been a revolutionary in terms of the scope and depth of response now sought in treating rheumatic diseases. Those best characterized are TNFi, IL-6Ri, rituximab and abatacept, all of which bring about improvement in clinical signs and symptoms, and variously mitigate articular destruction and improve function. Application of drugs developed for other indications but now being tested in rheumatic diseases has raised interest in the possible value of agents such as ustekinumab (inhibiting IL-12/23), and several antibodies that block the biology of IL-17. The latter appear to exhibit especial benefits in the spondylo arthropathies and psoriatic variants of inflammatory arthropathy. Other studies targeting GM-CSF receptor have similarly shown benefit in early studies in rheumatoid arthritis. The long promised possibility that chemokines and their receptors would deliver benefit now has some tentative evidence for benefit. In this presentation I shall describe the successes and failures of recent biologic therapeutic studies in the rheumatic disease, and wider inflammatory arena, and from this draw inferences as to new emerging understanding of the molecular taxonomy of the inflammatory diseases.

## Disclosure of interest

None declared.

